# 2472. Hospital Onset Antibiotic Resistant Infections among Patients without COVID-19 Diagnosis in Pre-Pandemic and Pandemic Periods

**DOI:** 10.1093/ofid/ofad500.2090

**Published:** 2023-11-27

**Authors:** James Baggs, Natalie McCarthy, Sujan Reddy, Hannah Wolford

**Affiliations:** CDC, atlanta, Georgia; CDC, atlanta, Georgia; CDC, atlanta, Georgia; CDC, atlanta, Georgia

## Abstract

**Background:**

In 2022, Centers for Disease Control and Prevention reported that hospital onset (HO) antibiotic resistant (AR) infections increased during the COVID-19 pandemic. To determine whether these increases were related only to patients diagnosed with COVID-19 or to changes in healthcare utilization and case-mix, we compared the risk of HO AR infections before and during the COVID-19 pandemic among hospitalized patients without a diagnosis of COVID-19.

**Methods:**

Using the PINC AI Healthcare Database, we included discharges from the pre-pandemic period (January 1, 2019-January 30, 2020) and the pandemic period (April 1, 2020-December 31, 2021), among facilities reporting microbiology data. We excluded discharges with a COVID-19 diagnosis, an inpatient COVID-19 diagnosis in the previous 90 days, or length of stay ≤ 3 days. Using a multivariable Cox proportional hazards model, we calculated hazard ratios (HR) comparing the risk of incident HO AR infections during the pre-pandemic and pandemic periods, adjusting for patient demographics, comorbidities, hospitalization in the previous 30 days, facility-level rate of community onset AR infections by discharge month, time-dependent intensive care unit stay and mechanical ventilation, facility characteristics, and inter-facility correlation.

**Results:**

There were 1,153,799 discharges from 280 facilities in the pre-pandemic period, and 1,470,063 discharges from 287 facilities in the pandemic period. The risk of HO ESBL, CRAsp, MDR Pseudomonas, and VRE among patients without COVID-19 was significantly elevated during the pandemic period (Figure 1). The risk of HO MRSA was significantly decreased during the pandemic period.

**Figure:**
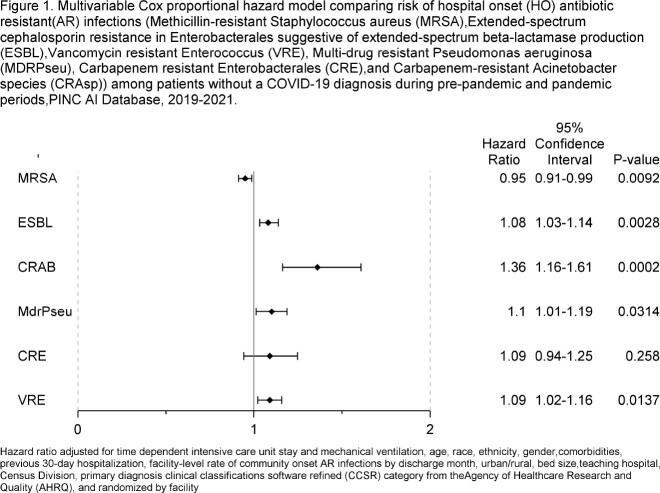

**Conclusion:**

The risk of HO AR infections, except for MRSA and CRE, was significantly increased in patients not diagnosed with COVID-19 during the pandemic period even after controlling for demographics, clinical, and facility characteristics. Understanding potential drivers for these increases, such as infection prevention barriers, is important for pandemic preparedness and healthcare resiliency.

**Disclosures:**

**All Authors**: No reported disclosures

